# Age effect on bone mineral density changes in breast cancer patients receiving anastrozole: results from the ARBI prospective clinical trial

**DOI:** 10.1007/s00432-012-1233-z

**Published:** 2012-05-03

**Authors:** Christos Markopoulos, Evagelos Tzoracoleftherakis, Dimitrios Koukouras, Basileios Venizelos, Vasilios Zobolas, John Misitzis, Grigorios Xepapadakis, Helen Gogas

**Affiliations:** 1 Hellenic Society of Breast Surgeons, 6 Eslin Street, 11523 Athens, Greece; 2 8 Iassiou Street, 11521 Athens, Greece

**Keywords:** Breast cancer, Aromatase inhibitors, Anastrozole, Bisphosphonates, Risedronate, Bone mineral density

## Abstract

**Purpose:**

We investigated whether age at anastrozole (A) initiation influences the effect of treatment on bone mineral density (BMD). We conducted a post hoc analysis of the dataset of Arimidex Bone Mass Index Oral Bisphosphonates prospective trial, studying the effect of risedronate (R) on BMD of postmenopausal, early breast cancer patients receiving A.

**Methods:**

Patients were stratified into those with normal BMD or mild osteopenia (*T* > −2) receiving A-only and patients with mild or severe osteopenia (*T* ≤ −2) or osteoporosis (*T* < −2.5) receiving A and per os R (A + R). Depending on age on treatment initiation, patients were grouped into two age cohorts, above and below 65 years. BMD change in lumbar spine (LS) and hip (HP) was evaluated at 12 months. An analysis of patients with normal BMD at baseline was additionally performed.

**Results:**

Among patients receiving A-only, women ≤65 years were more likely to have a decrease in LS-BMD than older (*p* = 0.034). HP-BMD decrease at 12 months was not related to age (*p* = 0.182). In patients with mild or severe osteopenia or osteoporosis, treated with A + R, no age effect was observed for LS or HP (*p* = 0.099 and *p* = 0.939, respectively). Among patients with normal BMD at baseline, the age effect on LS-BMD change was more profound (*p* = 0.026).

**Conclusions:**

Our study suggests that younger postmenopausal women with normal BMD or mild osteopenia receiving A-only face an increased risk of bone loss in LS. Among patients with mild or severe osteopenia or osteoporosis treated with A + R, 12 months LS or HP BMD variations were configured regardless of age group.

## Introduction

The principle treatment options for postmenopausal patients with hormone receptor (HR)-positive breast cancer are third-generation aromatase inhibitors (AIs) and selective estrogen receptor modulators (SERMs). Adjuvant therapy with AIs has significant benefits over SERMs in terms of disease-free survival and safety (Rugo 2008; Baum et al. 2003; Coombes et al. 2004; Goss et al. 2005; Gibson et al. 2009; Howell et al. 2005; Markopoulos 2010), and it is recommended by International Scientific Associations (Burstein et al. 2010; Carlson et al. 2006; NCCN 2011; NICE 2009).

Breast cancer and treatment increases the risk of osteoporosis, falls, and fractures in women affected (Chen et al. 2005a, b, 2009; Kanis et al. 1999). AIs reduce systemic estrogen through 98 % inhibition of the aromatase enzyme (Geisler et al. 1996, 2002; Dixon et al. 2008), and this estrogen deficiency is associated with an accelerated bone loss. Bisphosphonates (BPs) are considered effective supportive therapy in the decrease in skeletal complications in different types of cancers, including breast cancer (Body et al. 1998; Aapro et al. 2008; Markopoulos et al. 2010). BPs inhibit bone resorption by interfering with osteoclast activation and by promoting osteoclast apoptosis (Body et al. 1998; Fleisch 2002; Ashcroft et al. 2003; Gnant and Eidtmann 2010). Clinical evidence supports their use as add-ons to AIs as a protective measure against osteoporosis (Gnant et al. 2007; Brufsky et al. 2009; Bundred et al. 2008; Lester et al. 2008; Van et al. 2010).

Our group recently published the results of the Arimidex Bone Mass Index and Oral Bisphosphonates (ARBI) multicenter, prospective, open-label study on the effect of the BP risedronate on BMD in postmenopausal, early breast cancer patients scheduled to receive anastrozole (Markopoulos et al. 2010). The ARBI study demonstrated that postmenopausal patients with normal BMD before starting anastrozole had a low risk of developing osteoporosis during the first 2 years of treatment. Furthermore, risedronate co-administration significantly increased BMD levels in patients with pre-treatment osteopenic to osteoporotic status (Markopoulos et al. 2010).

It is still not known, however, whether AIs affect bone density in the same way within different age groups of postmenopausal women. This is particularly interesting because estrogen deficiency leads to bone density decreases with age: every year, 1 of 3 women above 65 years has a fall and sustains fractures (ESHRE Capri Workshop Group 2010; Sambrook and Cooper 2006; Khosla et al. 1997), and 65 years of age is an indication for measuring BMD in osteoporosis guidelines (National Osteoporosis Foundation 2010; O’Neill et al. 2004; Papaioannou et al. 2010). Furthermore, recent research results indicate that younger patients on A treatment may face risk of increased bone-resorption rates (Powell et al. 2011).

In this context, we conducted this post hoc analysis of the ARBI dataset to determine the effect of age on anastrozole-induced bone loss.

## Patients and methods

### Study design

The ARBI study was multicenter, prospective, open-label study on the effect of the BP risedronate on BMD in postmenopausal, early breast cancer patients scheduled to receive anastrozole (*Trial registration*: ClinicalTrials.gov Identifier NCT00809484).

Patients were to have undergone primary surgery and chemotherapy (if indicated). Exclusion criteria were drug-induced menopause, evidence of metastatic bone disease, previous hip fractures or prostheses, bone metabolism disorders, untreated hypocalcemia, previous treatment with selective estrogen receptor modulators, hormone-replacement therapy, or bisphosphonates (BPs), and liver or renal dysfunction. All women received Anastrozole (ArimidexTM AstraZeneca, London, UK) 1 mg/day and were followed up for 24 months. All patients had to give informed consent prior to enrollment in the study. Full local ethics committee approval was successfully obtained in all sites recruiting patients for the study, and national ethics committee approval of the trial protocol was also obtained. The design and results of the ARBI trial have been previously reported (Markopoulos et al. 2010).

### Patient groups

A total of 213 postmenopausal patients with HR-positive breast cancer were enrolled into the ARBI study.

**Fig. 1:**
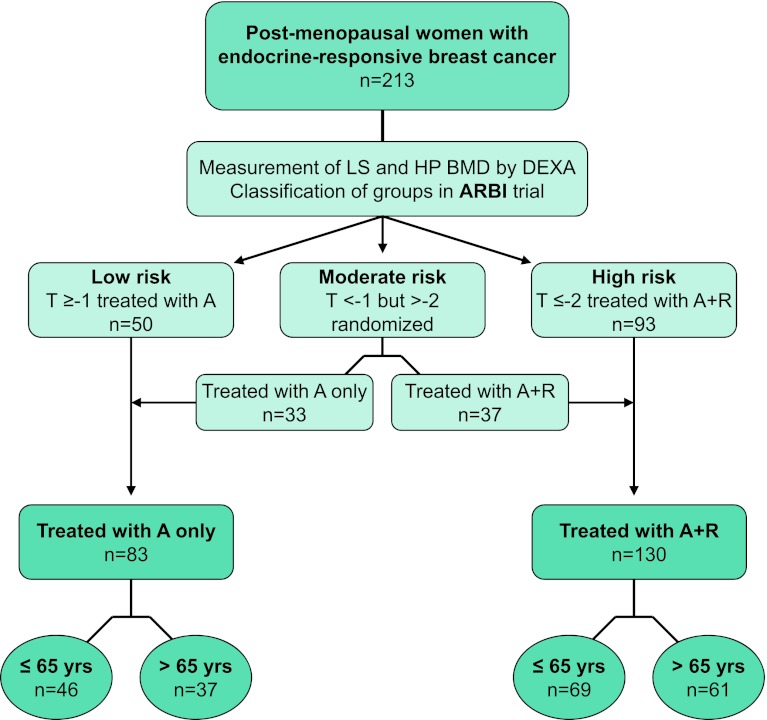
Schema of the post hoc age subgroup analysis. A, anastrozole; R, Risedronate; BMD, bone mineral density; DEXA, dual-energy X-ray absorptiometry; HP, hip; LS, lumbar spine

Participants were assigned to three risk groups (Fig. 1) for developing aromatase inhibitor-associated bone loss based on their baseline BMD T-score measured in lumbar spine (LS) and hip (HP):

- low risk with a normal *T*-score ≥−1 at both sites
- mild-to-moderate risk with *T*-score <−1 at either site and >−2 at both sites
- high risk with a *T*-score ≤−2 at either site.

Patients in low risk group (*n* = 50) with normal BMD at baseline (*T*-score ≥ −1 at both sites) received treatment with Anastrozole alone. The medium risk group (*n* = 70) was randomized to receive Risedronate in addition to Anastrozole (*n* = 37) or Anastrozole alone (*n* = 33), and Risedronate (35 mg/week) was administered to all high risk patients (*n* = 93).

Additionally, all patients on study received daily supplements of Vitamin D (400 IU/day) and Calcium (1,000 mg/day). This is because it is known that older, postmenopausal women are at increased risk of developing vitamin D insufficiency because they may have inadequate intakes of vitamin D as well as of calcium, and additionally skin cannot synthesize the vitamin efficiently by aging (Institute of Medicine, Food and Nutrition Board 2010). As calcium is necessary for maintaining bone health and vitamin D is important for the absorption of calcium from the stomach, supplements were given to prevent as possible the effect that their insufficient intake would have had on BMD and influence the effect of medicines under study.

### Assessments

The primary endpoint of the ARBI study was to investigate the effect of R in the randomized arms measured in both LS and HP at 12 months. BMD levels were evaluated at baseline before anastrozole administration, at 12, and at 24 months (secondary endpoint), by dual-energy X-ray absorptiometry (DEXA).

Patient demographic data, Eastern Cooperative Oncology Group (ECOG) Performance Status (Oken et al. 1982), and fracture history were recorded at baseline.

### Formulation of the post hoc statistical analysis

The aim of this unplanned subgroup analysis was to explore whether age at baseline differentiates the effect of A on BMD change at 12 months post-treatment initiation. Depending on age on treatment initiation, patients were grouped into two age cohorts above and below 65 years, since this is the threshold for measuring BMD in osteoporosis guidelines (National Osteoporosis Foundation 2010; O’Neill et al. 2004; Papaioannou et al. 2010).

In the present analysis, we stratified the ARBI study patients (low risk treated with A-only, moderate with or without R and high risk with R) into two groups according to the administration or not of Risedronate: (a) patients with normal BMD or mild osteopenia (*T* > −2) receiving A-only and (b) patients with mild or severe osteopenia (*T* ≤ −2) or osteoporosis (*T* < −2.5) receiving A and per os R (A + R). The classification of patients as described above is presented in Fig. 1.

The outcome measure of change at 12 months post-treatment initiation was calculated as the ratio 12 m/baseline and expressed as percentage. Comparisons between the age groups were performed using the *t* test. 95 % confidence intervals (CI) for changes as well as for the difference between changes are reported in all cases in order to facilitate the assessment of the clinical significance of the findings.

Since baseline BMD levels may affect the temporal variation of BMD, we also performed a covariance analysis using baseline BMD as a confounding factor for the effect of age to account for possible predisposition bias caused by baseline imbalances on BMD (interaction effect). All statistical tests were evaluated at the 5 % level of significance.

## Results

Of all patients, 54.5 % (116/213) were 65 years or younger and 45.5 % (97/213) were older (Table 1). The ECOG status was 0 for nearly all ≤65-year-old patients (% range: 97.9–100.0) and for most >65-year-old patients (% range: 75.0–77.0). Seven patients had sustained traumatic fractures 3–56 years before study enrollment, none of the LS or HP.

**Table 1:** Patient baseline characteristics by age group and treatment strata

	A		A + R		Total	
	≤65	>65	≤65	>65	≤65	>65
Age (years)						
N	47	36	69	61	116	97
Mean ± SD	57 ± 4.7	71 ± 4.6	58 ± 4.1	72 ± 4.5	58 ± 4.4	72 ± 4.6
BMD LS						
Mean ± SD	1.04 ± 0.12	1.02 ± 0.14	0.84 ± 0.14	0.80 ± 0.16	0.91 ± 0.16	0.88 ± 0.19
BMD HP						
Mean ± SD	0.88 ± 0.13	0.84 ± 0.10	0.76 ± 0.09	0.71 ± 0.11	0.81 ± 0.12	0.76 ± 0.12
BMI						
Mean ± SD	28.76 ± 5.51	29.57 ± 3.85	26.89 ± 5.02	28.62 ± 4.22	27.65 ± 5.28	28.97 ± 4.09

	N (%)					
ECOG status						
0	46 (97.9)	27 (75.0)	69 (100.0)	47 (77.0)	115 (99.1)	74 (76.3)
1	1 (2.1)	9 (25.0)	0 (0.0)	14 (23.0)	1 (0.9)	23 (23.7)
Fracture history						
No	41 (87.2)	35 (97.2)	67 (97.1)	54 (88.5)	108 (93.1)	89 (91.8)
Yes^a^	3 (6.4)	0 (0.0)	1 (1.4)	3 (4.9)	4 (3.4)	3 (3.1)
Not reported	3 (6.4)	1 (2.8)	1 (1.4)	4 (6.6)	4 (3.4)	5 (5.2)

*A* anastrozole, *R* risedronate, *BMD* bone mineral density, *BMI* body mass index, *ECOG* eastern cooperative oncology group, *SD* standard deviation

^a^Traumatic fractures only; between 3 and 56 years before enrollment in the study; none in the hip (HP) or lumbar spine (LS)

**Fig. 2:**
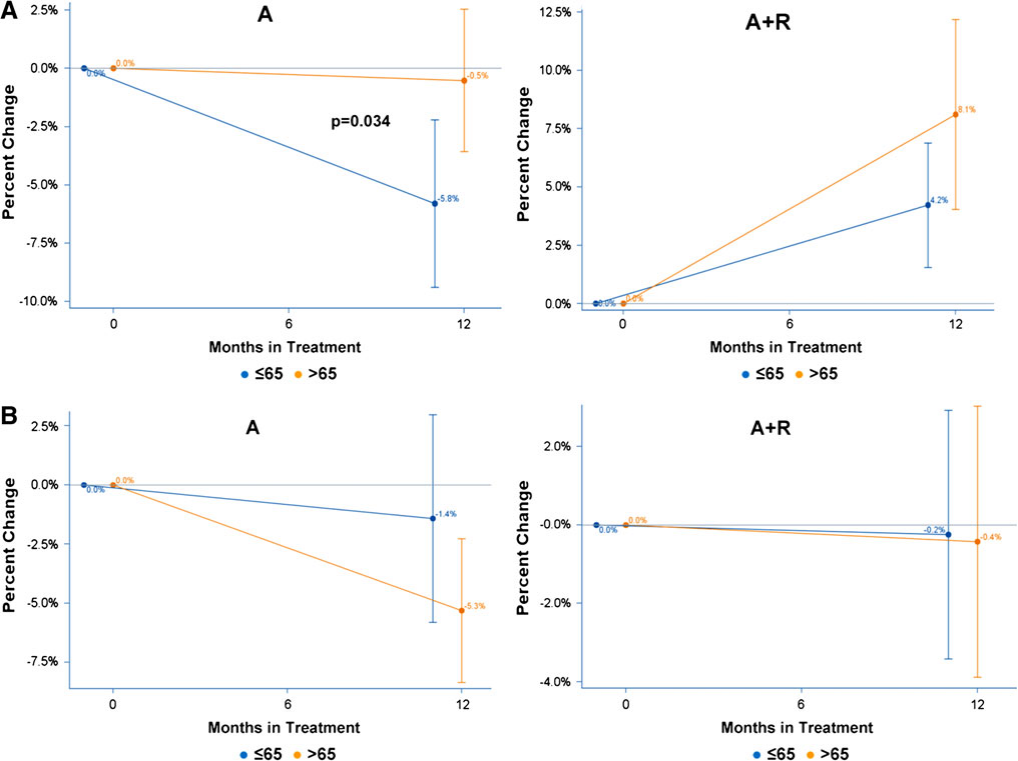
**a** Average BMD change from baseline at lumbar spine (*LS*) by age group, in patients on Anastrozole-only (A) and on Anastrozole plus Risedronate (A + R). **b** Average BMD change from baseline at hip (HP) by age group, in patients receiving Anastrozole-only (A) or Anastrozole plus Risedronate (A + R)

Figure 2a shows the average percent BMD change from baseline and the 95 % CI for LS and HP in patients with normal BMD or mild osteopenia at baseline receiving treatment with A-only. Mean BMD percent change in LS was −5.8 % (95 % CI: −9.5 %, −2.1 %) for patients ≤65 and −0.5 % (95 % CI: −3.7 %, 2.6 %) for patients >65. The difference in density loss of 5.3 % (95 % CI: 0.4 %, 10.1 %) between the age groups was statistically significant (*p* = 0.034, Table 1). Mean BMD percent change in HP was −1.4 % (95 % CI: −5.9 %, 3.0 %) for patients ≤65 and −5.3 % (95 % CI: −8.5 %, −2.2 %) for patients >65. The difference in density loss of 3.9 % (95 % CI: −1.9 %, 9.7 %) between the age groups was not statistically significant (*p* = 0.182, Table 1).

In patients with mild or severe osteopenia or osteoporosis receiving A + R (Fig. 2B), mean BMD percent change in LS was +4.2 % (95 % CI: 1.5 %, 6.9 %) for patients ≤65 and +8.1 % (95 % CI: 4.0 %, 12.2 %) for patients >65. The difference in density gain of 3.9 % (95 % CI: −0.7 %, 8.5 %,) between the age groups was not statistically significant (*p* = 0.099, Table 1). Mean BMD percent change in HP was −0.2 % (95 % CI: −3.4 %, 2.9 %) for patients ≤65 and −0.4 % (95 % CI: −3.9 %, 3.1 %) for patients >65. The difference in density loss of 0.2 % (95 % CI: −4.5 %, 4.9 %) between the age groups was not statistically significant (*p* = 0.939, Table 1).

**Fig. 3:**
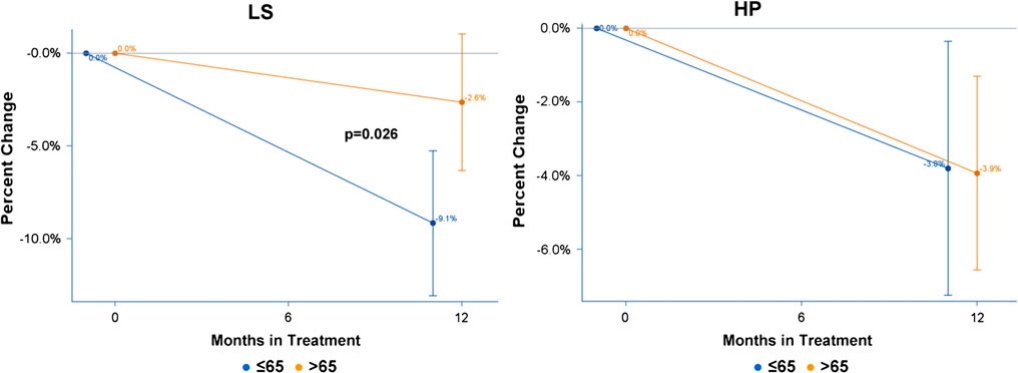
Average BMD change at lumbar spine (*LS*) and hip (*HP*) by age group, in 50 patients with normal BMD at baseline, receiving A-only

Figure 3 shows the average percent BMD change from baseline and the 95 % CI for LS and HP for the substratum of 50 patients with normal BMD at baseline. Mean BMD percent change in LS was −9.1 % (95 % CI: −13.2 %, −5.1 %) for patients ≤65 and −2.6 % (95 % CI: −6.6 %, 1.3 %) for patients >65. The difference in density loss of 6.5 % (95 % CI: 0.8 %, 12.2 %) between the age groups was statistically significant (*p* = 0.026, Table 2). Mean BMD percent change in HP was −3.8 % (95 % CI: −7.4 %, −0.2 %) for patients ≤65 and −3.9 % (95 % CI: −6.8 %, −1.1 %) for patients >65. The difference in density loss of 0.1 % (95 % CI: −4.7 %, 5.0 %) between the age groups was not statistically significant (*p* = 0.957, Table 2).

**Table 2:** Average BMD change from baseline (95 % CI) by age group at 12 months

	≤65	>65	Difference	*p* value
Based on treatment				
LS				
A	−5.8 % (−9.5 %, −2.1 %)	−0.5 % (−3.7 %, 2.6 %)	5.3 % (0.4 %, 10.1 %)	0.034
A + R	4.2 % (1.5 %, 6.9 %)	8.1 % (4.0 %, 12.2 %)	3.9 % (−0.7 %, 8.5 %)	0.099
HP				
A	−1.4 % (−5.9 %, 3.0 %)	−5.3 % (−8.5 %, −2.2 %)	3.9 % (−1.9 %, 9.7 %)	0.182
A + R	−0.2 % (−3.4 %, 2.9 %)	−0.4 % (−3.9 %, 3.1 %)	0.2 % (−4.5 %, 4.9 %)	0.939
Patients (*N* = 50) with normal BMD at baseline, receiving A alone				
LS	−9.1 % (−13.2 %, −5.1 %)^a^	−2.6 % (−6.6 %, 1.3 %)^b^	6.5 % (0.8 %, 12.2 %)	0.026
HP	−3.8 % (−7.4 %, −0.2 %)^c^	−3.9 % (−6.8 %, −1.1 %)^d^	0.1 % (−4.7 %, 5.0 %)	0.957

*LS* lumbar spine, *HP* hip, *A* anastrozole, *R* risedronate, *BMD* bone mineral density, *CI* confidence interval

^a^ *N* = 20; ^b^ *N* = 14; ^c^ *N* = 21; ^d^ *N* = 14

Covariance analysis examined whether the effect of age is truly caused by differences in baseline BMD (Table 3). The age effect on LS changes at 12 months in patients with normal BMD or mild osteopenia at baseline remained statistically significant even after adjustment by baseline BMD values (*p* = 0.0295). Moreover, the estimate of the adjusted difference was almost identical to the unadjusted (−5.147 and −5.3 %, respectively). The negative effect of baseline BMD value on LS changes at 12 months is statistically significant in both treatment groups (A and A + R, *p* value = 0.0098 and *p* value < 0.001, respectively). This means that patients with higher baseline BMD levels are more likely to present larger loss or smaller increase at 12 months compared with patients with smaller baseline BMD levels. However, regarding HP, no statistically significant associations were detected (Table 3; results are shown without the interaction effect that was not significant).

**Table 3:** Covariance analysis, age effect on the percent change from baseline adjusted by baseline BMD values

Effect	LS		HP	
	Estimate	*p* value	Estimate	*p* value
A				
Age (≤ 65 vs. > 65)	−0.05147	0.0295	0.03734	0.1996
Baseline BMD	−0.2551	0.0098	−0.2029	0.0997
A + R				
Age (≤ 65 vs. > 65)	0.02446	0.2649	−0.00425	0.8595
Baseline BMD	−0.2917	<0.0001	−0.05986	0.4432

*LS* lumbar spine, *HP* hip, *A* anastrozole, *R* risedronate, *BMD* bone mineral density

## Discussion

We conducted this post hoc analysis of the ARBI dataset to evaluate the difference in BDM changes between age groups. Our results indicate that women >65 years are more likely to experience larger increases or smaller decreases in their LS-BMD levels than women ≤65 years. In HP, no statistically significantly differences were recorded between age groups.

In patients with normal BMD or mild osteopenia at baseline receiving treatment with A-only, our observation of a systematic deterioration in BMD levels in patients ≤65 years could be possibly attributed to greater skeletal changes occurring in younger patients when receiving anastrozole. This greater change in BMD when anastrozole is administered to younger women was shown by Powell et al. 2011. They compared the levels of the bone-resorption marker uNTx (urinary cross-linked N-telopeptide of type I collagen) in newly diagnosed women with breast cancer who were receiving anastrozole and were above or below the age of 70 years with that of healthy women of the same age group (<70 or ≥70 years). The group of younger women had statistically significantly higher levels of the bone-resorption marker compared with their healthy counterparts, while the older age group had similar levels compared with healthy women. The uNTx levels in younger women on anastrozole were similar to those in elderly women, both healthy and on anastrozole. In younger women, uNTx exceeded normal levels but not in older women. This could be attributed to higher levels of free estradiol in younger, postmenopausal women, which allows for more marked effects with the aromatase inhibitors (Powell et al. 2011). Supporting data from the literature show that there is a decline in free estradiol levels with aging (Bjornerem et al. 2004); this seems to be caused by a rise of sex hormone globulin levels found in elderly women rather than due to decreased levels of total estradiol in this age group (Sharp et al. 1996; Laughlin et al. 2000). However, differences in the degree of BMD change caused by anastrozole between elderly breast cancer patients and their younger counterparts could also be due to some extend to a different sensitivity of hormone receptors to circulating estradiol, developed by aging. This hypothesis needs further investigation.

Comparing the anastrozole effect on LS and HP, in the ATAC (Eastell et al. 2006) as well as in the SABRE trial (Van Poznak et al. 2010), anastrozole significantly reduced BMD of both LS and HP; notably, in both trials, patients showed a greater BMD loss in the lumbar region (LS) than at femoral sites (HP). In our study, results are in the same direction with the above trials, although changes in HP were not statistically significant in our study population; anastrozole had a negative effect on femoral BMD as well, whereas risedronate was shown that can prevent this BMD loss (Table 2; Fig. 2a). This difference in the degree of BMD change according to sites (LS and HP) could be attributed to different sensitivity of receptors to AI-induced estrogen deprivation of lumbar region and femoral sites with advancing age.

Crivellari et al. 2008 investigated differences in response to letrozole treatment and adverse events by age groups but did not assess bone marker profiles, only bone fractures, which were similar across different age groups. The ATAC trial (Arimidex Tamoxifen Alone or in Combination) showed greater BMD losses in anastrozole-treated women who experienced menopause within the last 4 years than in those who were more than 4 years postmenopausal (Eastell et al. 2006). A subsequent analysis of the same study for the investigation of potential risk factors for joint symptoms showed no detectable effect of age (Sestak et al. 2008).

Our literature search did not retrieve any other studies comparing the impact of AIs on bone marker profile measurements depending on the age of the patient. Our results and those published by Powell et al. 2011 indicate the need for age group comparison of bone markers and further evaluation of the impact the AIs have on different age groups of patients. AIs have been found to have higher efficacy than Tamoxifen (Rugo 2008; Baum et al. 2003; Coombes et al. 2004; Goss et al. 2005; Gibson et al. 2009; Howell et al. 2005; Markopoulos 2010), and there is a general recommendation for the administration of an AI at some point during the adjuvant hormonal treatment of postmenopausal patients with hormone receptor positive early breast cancer (Burstein et al. 2010). However, there is concern about the negative effect they have on BMD in contrast to Tamoxifen and this is important for both, younger postmenopausal women losing bone mass due to recent menopause and aging, and elderly women often having already osteopenia-osteoporosis but different life expectancy. Therefore, it is very important to explore possible differences in the effect that AIs might have on BMD according to age of patients and to BMD status before the administration of an AI, so appropriate supportive measures could be taken in clinical practice.

Moreover, our findings render the general perception to feel “safe” considering “bone loss” when starting adjuvant treatment with an AI in patients having normal BMD before treatment, questionable. Maybe young, postmenopausal patients starting AI treatment should be followed for BMD changes, especially in LS, despite normal BMD at baseline.

Notably, our results are limited by the fact that this is an unplanned subgroup analysis of the ARBI trial, and they were not powered to investigate such hypothesis. Despite this, they do indicate that a future study aiming to explicitly address this specific issue of age-related BMD changes in postmenopausal breast cancer patients on adjuvant treatment with AIs is justified.

In conclusion, our results suggest that among patients with normal BMD or mild osteopenia receiving A-only, younger women face an increased risk of BMD loss in LS 12 months post-treatment initiation, especially if they present with normal BMD. Among patients with mild or severe osteopenia or osteoporosis, AI treatment side effect on BMD is not related to age group.

## Abbreviations

A: Anastrozole
AI: Aromatase inhibitor
ARBI: Arimidex bone mass index and oral bisphosphonates
ATAC: Arimidex, tamoxifen alone or in combination
BMD: Bone mineral density
BP: Bisphosphonate
DEXA: Dual-energy X-ray absorptiometry
ECOG: Eastern cooperative oncology group
HP: Hip
LS: Lumbar spine
R: Risedronate
SD: Standard deviation
SERMs: Selective estrogen receptor modulators
T: T-score
WHO: World Health Organization
